# Survival benefit associated with first-line androgen receptor pathway inhibitors for de novo metastatic castration-sensitive prostate cancer

**DOI:** 10.1038/s41391-025-01000-8

**Published:** 2025-07-25

**Authors:** Stephen J. Freedland, Agnes Hong, Nader El-Chaar, Amanda M. De Hoedt, Janet Kim, Claire Evans, Joshua A. Parrish, Maelys Touya, Krishnan Ramaswamy, Lin Gu, Thomas J. Polascik

**Affiliations:** 1 Section of Urology, Durham VA Health Care System, Durham, NC USA; 2 Department of Urology, Samuel Oschin Comprehensive Cancer Institute, Cedars-Sinai Medical Center, Los Angeles, CA USA; 3 Pfizer Inc., New York, NY USA; 4 Astellas Pharma Inc., Northbrook, IL USA; 5 Duke Cancer Institute, Duke University School of Medicine, Durham, NC USA

**Keywords:** Cancer therapy, Prostate cancer

## Abstract

**Background:**

Limited real-world data exist on the effectiveness of treatment intensification (TI) with androgen receptor pathway inhibitors (ARPI) in de novo metastatic castration-sensitive prostate cancer (mCSPC). This study compared outcomes of TI or first-generation nonsteroidal antiandrogens (NSAAs) to androgen-deprivation therapy (ADT) alone in US patients with de novo mCSPC.

**Methods:**

Veterans Affairs patients with de novo mCSPC (February 2018–June 2020) confirmed via chart review were grouped into ADT alone, ADT + NSAAs, or ADT + ARPI cohorts using predefined recruitment quotas. Outcomes included inverse probability of treatment weighting (IPTW)-adjusted overall survival (OS), progression to metastatic castration-resistant prostate cancer (mCRPC), and prostate-specific antigen (PSA) response.

**Results:**

A total of 384 patients were identified (ADT alone: 163, ADT + NSAA: 101, ADT + ARPI: 120). Median follow-up was 37.2, 38.1, and 34.8 months for ADT alone, ADT + NSAA, and ADT + ARPI, respectively. Compared with ADT alone, ADT + ARPI showed significantly better OS (HR [95% CI]: 0.61 [0.43 to 0.87], *p* = 0.007), lower risk of progression to mCRPC (0.46 [0.33 to 0.66], *p* < 0.001), and higher PSA response rate (PSA decline of ≥50% and ≥90% from baseline, and to <0.2 ng/mL and <0.1 ng/mL any time during first-line treatment; all *p* < 0.05). Outcomes with ADT + NSAA did not differ from ADT alone. ADT + ARPI was the most common second-line mCSPC and first-line mCRPC treatment.

**Conclusions:**

First-line ADT + ARPI was associated with significantly improved outcomes vs ADT alone in de novo mCSPC. These real-world results align with the benefits demonstrated in trials, supporting integration of TI with ARPIs into clinical practice to improve survival outcomes in patients with de novo mCSPC.

## Introduction

Prostate cancer (PC) is the most common cancer in men and the second leading cause of cancer death in the United States (US) [1]. Approximately 5−10% of patients with PC present with de novo metastatic castration-sensitive PC (mCSPC) at time of diagnoses [2]. These patients have an aggressive disease course with quick progression to castration resistance and worse overall survival (OS) compared with those who have primary progressive disease [3].

Androgen deprivation therapy (ADT) has been a mainstay of mCSPC treatment since the 1940s, with clinical benefits observed with or without nonsteroidal antiandrogens (NSAAs). In particular, the addition of NSAAs such as flutamide, nilutamide, or bicalutamide to ADT has shown modest improvements in progression and survival outcomes [4–9]. Although numerous treatment options have been developed for the treatment of mCSPC over the last several decades, none are able to cure advanced PC on their own. In addition, although the majority of patients with mCSPC initially respond to these treatments, most patients go on to develop hormone resistance, which is associated with a poor prognosis and a median survival time of approximately 3 years [10, 11]. In an effort to improve clinical outcomes for patients with mCSPC and delay the development of resistance, multiple clinical trials have been conducted over the last decade to evaluate the oncological benefit of new therapeutic agents and treatment regimens. These trials have demonstrated improved survival in patients with mCSPC who receive treatment intensification (TI), wherein ADT is used in combination with docetaxel and/or androgen receptor pathway inhibitors (ARPIs) such as abiraterone, apalutamide, darolutamide, and enzalutamide, compared with patients who receive ADT alone [10, 12–16]. In addition to clinical benefit over ADT alone, TI with ADT + ARPIs has also shown superiority over treatment regimens that combine ADT with NSAAs, suggesting that TI with ARPIs can be particularly beneficial [17]. Despite robust clinical trial evidence and guideline recommendations [18, 19], rates of TI remain low, with less than one-third of patients with mCSPC receiving first-line life-prolonging treatment regimens [11, 20–23]. Numerous real-world studies across US healthcare systems have shown that most patients continue to receive ADT alone or with first-generation NSAA therapies [11, 20–23].

To increase the adoption of TI in patients with mCSPC, it is critical to demonstrate the real-world effectiveness of ARPIs and validate the clinical trial survival improvements associated with first-line TI. Few real-world studies have reported survival outcomes of TI with ARPI vs ADT alone for de novo mCSPC [11, 21, 23, 24]. Corsini et al. found that TI with ARPI or chemotherapy led to a clinically meaningful increase in survival in patients with de novo mCSPC between 2008 and 2020 [21]. In another real-world study in Japan, first-line TI with docetaxel or abiraterone showed better OS vs ADT alone or combined androgen blockage in patients with high-volume mCSPC [23]. Notably, none of these studies were conducted in the US, highlighting a literature gap for contemporary real-world data on outcomes associated with first-line TI with ARPI compared to ADT alone in the US population.

This study assessed real-world patient characteristics, subsequent treatment patterns, and outcomes in patients with de novo mCSPC initiating first-line treatment with ADT + ARPI or ADT + NSAA compared with ADT alone among men treated in the US Veterans Affairs (VA) healthcare system.

## Materials and methods

### Study design and data source

This was a retrospective chart review study capturing data from the VA’s electronic health records system from February 1, 2017–March 11, 2023. The cohort was identified via the Corporate Data Warehouse within the Veterans Affairs Informatics and Computing Infrastructure (VINCI). All relevant data elements were captured from the individual-level medical records containing demographics, diagnosis, treatments, radiology reports, and laboratory values. This study received Institutional Review Board and Research and Development approval from the Durham VA Health Care System with waiver of informed consent (IRB#1827). This study was conducted in accordance with the Declaration of Helsinki.

### Patient identification

Adult patients (≥18 years of age) were included if they had a de novo mCSPC diagnosis, confirmed by clinical chart review, between February 1, 2018 and June 30, 2020 and received first-line ADT alone, ADT + NSAA, or ADT + ARPI ± NSAA (hereafter referred to as ADT + ARPI). This time frame was chosen to align with the approval of abiraterone for use in mCSPC [13]. Key exclusion criteria were treatment with cabazitaxel, docetaxel, mitoxantrone, carboplatin, cisplatin, oxaliplatin, radium-223, sipuleucel-T, radical prostatectomy, or prostate-directed radiation therapy any time before the index date, and other cancers (excluding non-melanoma skin cancer) during the baseline period. The index date was the date of ADT initiation.

Patients with de novo mCSPC who met the selection criteria were categorized into three cohorts:

- ADT alone: Patients who did not receive other treatments with ADT or were treated with NSAA for <3 months (treatment of testosterone flare).
- ADT + NSAA (nilutamide, bicalutamide, flutamide): Patients who received ADT and NSAA for ≥3 months after initial ADT date.
- ADT + ARPI: Patients who received an ARPI (enzalutamide or abiraterone) within 3 months after or within 30 days before ADT initiation.

The total target recruitment quotas were set for up to 400 patients, with allocations of 160 patients in ADT alone (80 Black and 80 White), 120 in ADT + NSAA (60 Black and 60 White), and 120 in ADT + ARPI (25–50% Black and 50–75% White) cohorts to ensure representation of each treatment regimen and across the two races. Given the limited percent of non-White and non-Black patients within the VA, analyses were limited to White and Black patients with mCSPC. Moreover, to identify patients with longer follow-up and optimize the identification of patients on ADT + ARPI who could have short follow-up given the slow uptake of TI, patients were randomly selected, ensuring that no more than 20% were diagnosed between January 1, 2020 and June 30, 2020.

### Study outcomes

The study outcomes included patient characteristics, subsequent treatment patterns, OS, progression to metastatic castration-resistant prostate cancer (mCRPC), and proportion of patients with prostate-specific antigen (PSA) decline of ≥50% and ≥90% from baseline, and to <0.2 ng/mL and <0.1 ng/mL, at any time during first-line treatment. The duration of first-line treatment was the time from the index date to the date of progression to mCRPC, discontinuation of first-line treatment, addition of a new systemic therapy to first-line treatment, death, or date of most recent follow-up, whichever came first. Subsequent treatment lines were stratified by disease stage of mCSPC or mCRPC. Second-line therapy for mCSPC included any change in therapy after first-line treatment while the patient remained castration-sensitive (no PSA rise or development of metastasis while on continuous ADT). In addition, this study captured the last regimen observed while de novo mCSPC through the first mCRPC treatment line, defined as any systemic therapy observed on or after the date of castration resistance. OS was measured as the duration from the index date to death from any cause. Living patients were censored at the most recent follow-up (MRFU) date. Time to mCRPC was from the index date to progression to mCRPC. Patients at MRFU without progression were censored. Progression to mCRPC and PSA response criteria are included in Supplementary Table 1.

### Statistical analyses

All study variables were summarized using descriptive statistics, with means and standard deviations (SD) or medians and interquartile ranges (IQR) for continuous variables, and frequencies and percentages for categorical variables. The standardized mean difference (SMD) was calculated for each baseline variable.

Multivariable analysis with inverse probability of treatment weighting (IPTW)-adjusted hazard ratios (HR) and 95% confidence intervals (CI) were estimated using Cox proportional hazards models for OS and time to mCRPC. IPTW-adjusted incidence rate ratios (IRR) and the associated 95% CI of the proportion of patients with PSA decline of ≥50% and ≥90%, and to <0.2 ng/mL and <0.1 ng/mL at any time during first-line treatment were estimated using Poisson regression, with the duration of first-line treatment included in the model to account for unequal follow-up time for patients. IPTW was calculated using a logistic regression, with the type of first-line treatment as the dependent variable and the covariates as independent variables (refer to Supplementary methods for detailed list of variables). The unadjusted results are included in Supplementary Table 2. Due to violating the Cox proportional HR assumption when fitting the regression of time to mCRPC among the three first-line treatment cohorts, a separate analysis comparing time to mCRPC between ADT + ARPI and ADT alone was conducted.

## Results

### Demographic and clinical characteristics

A total of 384 patients with de novo mCSPC were identified (ADT alone: *n* = 163; ADT + NSAA: *n* = 101; and ADT + ARPI: *n* = 120) (Supplementary Fig. 1). Although target recruitment quotas were implemented, there were a higher proportion of White patients across the three treatment cohorts (Table 1). Compared with patients receiving ADT alone, those receiving ADT + ARPI were younger, had a lower Charlson Comorbidity Index (CCI), and were more likely to have aggressive disease (high-volume disease, Gleason score of 9–10, and higher median baseline PSA) (Table 1). Patients receiving ADT + NSAA were of similar age to those receiving ADT alone, had a lower CCI and baseline median PSA but were more likely to have high-volume disease and Gleason score of 8 (Table 1).

**Table 1:** Demographic and clinical characteristics of patients with de novo mCSPC.

Patient characteristics	ADT alone (*n* = 163)	ADT + NSAA (*n* = 101)	ADT + ARPI^a^ (*n* = 120)
Race, *n* (%)			
Black	66 (41)	28 (28)^b^	38 (32)^b^
White	97 (60)	73 (72)^b^	82 (68)
Age (years) at index date, mean (SD)	76.4 (9.9)	76.9 (9.9)	70.6 (10)^b^
Region, *n* (%)			
South	70 (43)	41 (41)	43 (36)^b^
Midwest	25 (15)	23 (23)^b^	31 (26)^b^
West	34 (21)	20 (20)	27 (23)
Northeast	33 (20)	15 (15)^b^	18 (15)^b^
Puerto Rico	1 (1)	2 (2)	1 (1)
Year of mCSPC diagnosis, *n* (%)			
2018	66 (41)	38 (38)	36 (30)^b^
2019	81 (50)	50 (50)	68 (57)^b^
2020	16 (10)	13 (13)	16 (13)^b^
Pre-index treatment, *n* (%)	83 (51)	54 (54)	84 (70)^b^
Radiation therapy	9 (6)	1 (1)	3 (3)
Chronic corticosteroid use	78 (48)	54 (54)^b^	82 (68)^b^
Days from first PC diagnosis to mCSPC, median (IQR)	0 (0–14)	0 (0–4)	0 (0–16)
Days from mCSPC diagnosis to first-line initiation, median (IQR)	25 (9–43)	20 (9–34)	24 (9–37)
Grade group at PC diagnosis, *n* (%)			
Grade group 1 = Gleason 6 or fewer	3 (3)	1 (2)	0 (0)^b^
Grade group 2 = Gleason 3 + 4 = 7	2 (2)	2 (4)	3 (3)
Grade group 3 = Gleason 4 + 3 = 7	11 (12)	3 (6)	4 (4)^b^
Grade group 4 = Gleason 8	23 (26)	18 (33)^b^	27 (27)
Grade group 5 = Gleason 9–10	51 (57)	31 (57)	66 (66)^b^
Unknown	73	46	20
PSA (ng/mL) at index date, median (IQR)	121.6 (29.6–435.0)	92.8 (26.5–310.3)	162.7 (40.0–426.7)
Disease volume, *n* (%)			
High	82 (50)	63 (62)^b^	80 (67)^b^
Low	81 (50)	38 (38)^b^	40 (33)
CCI score^c^, mean (SD)	4.0 (3)	4.9 (3)^b^	3.7 (3)^b^
Baseline comorbidities, *n* (%)^d^			
Hypertension	129 (79)	86 (85)^b^	83 (69)^b^
Arrhythmia	55 (34)	39 (39)^b^	36 (30)
Chronic obstructive pulmonary disease	51 (31)	40 (40)^b^	45 (38)^b^
Hyperlipidemia	113 (70)	77 (76)^b^	82 (68)
Stroke	16 (10)	5 (5)^b^	6 (5)^b^
Acute coronary syndrome	10 (6)	6 (6)	6 (5)
Angina pectoris	16 (10)	12 (12)	14 (12)
Myocardial infarction	26 (16)	13 (13)	9 (8)^b^
Congestive heart failure	31 (19)	20 (20)	18 (15)^b^
Diabetes	65 (40)	43 (43)	48 (40)
Lower-extremity arterial occlusive disease	12 (7)	11 (11)^b^	14 (12)^b^

*ADT* androgen-deprivation therapy, *ARPI* androgen receptor pathway inhibitor, *CCI* Charlson Comorbidity Index, *mCSPC* metastatic castration-sensitive prostate cancer, IQR interquartile range, *NSAA* nonsteroidal antiandrogen, *PC* prostate cancer, *PSA* prostate-specific antigen, *SD* standard deviation, *SMD* standardized mean difference.

^a^Statistically significant as indicated by SMD (100 × actual SMD) > 10% vs ADT alone.

^b^ARPI included abiraterone acetate in 98 patients and enzalutamide in 22 patients.

^c^Solid cancer was not included in the CCI score.

^d^Only the top four comorbidities are presented.

### Treatment duration

The median (IQR) first-line treatment duration was longer for patients who received ADT + ARPI (20.3 [7.9─29.6] months) compared with those who received ADT alone (7.9 [4.8─17.4] months) or ADT + NSAA (9.5 [4.3─13.6] months).

### Overall survival

**Fig. 1:**
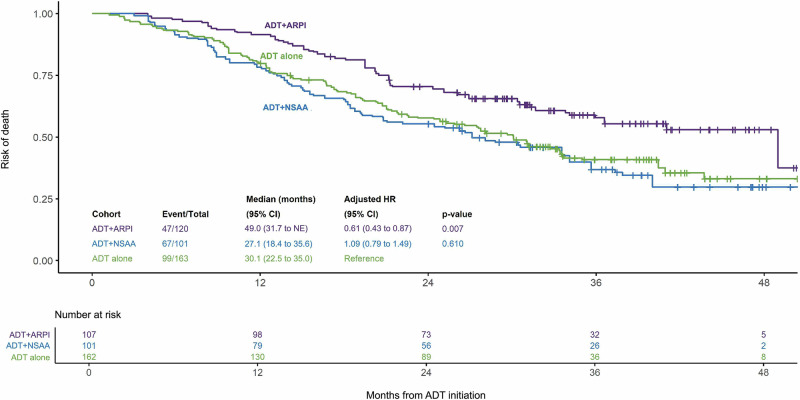
IPTW-adjusted OS comparison among patients with de novo mCSPC. ADT androgen-deprivation therapy, ARPI androgen receptor pathway inhibitor, CI confidence interval, HR hazard ratio, IPTW inverse probability of treatment weighting, mCSPC metastatic castration-sensitive prostate cancer, NE not estimable, NSAA nonsteroidal antiandrogen, OS overall survival.

The median (IQR) follow-up time was similar between the ADT alone (37.2 [31.9 to 42.4] months) and ADT + NSAA (38.1 [31.3 to 46.1] months) cohorts and slightly shorter for the ADT + ARPI cohort (34.8 [29.6 to 41.3] months). The adjusted median OS was 49.0 months for the ADT + ARPI compared with 27.1 and 30.1 months for the ADT + NSAA and ADT alone cohorts, respectively. Patients on ADT + ARPI had a 39% lower risk of death (adjusted HR: 0.61 [95% CI: 0.43 to 0.87]; *p* = 0.007), whereas those on ADT + NSAA (1.09 [0.79 to 1.49]; *p* = 0.610) had a similar risk of death compared with patients on ADT alone (Fig. 1).

### Progression to mCRPC

**Fig. 2:**
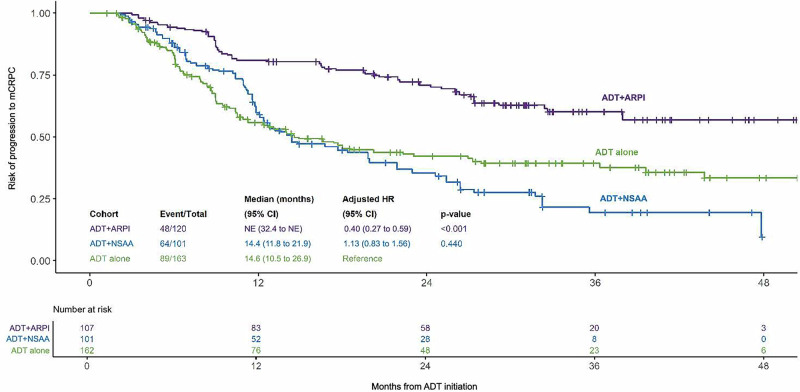
IPTW-adjusted time to mCRPC progression among patients with de novo mCSPC. ADT androgen-deprivation therapy, ARPI androgen receptor pathway inhibitor, CI confidence interval, HR hazard ratio, IPTW inverse probability of treatment weighting, mCRPC metastatic castration-resistant prostate cancer, mCSPC metastatic castration-sensitive prostate cancer, NE not estimable, NSAA nonsteroidal antiandrogen, OS overall survival, PSA prostate-specific antigen.

A total of 201 (52%) patients progressed to mCRPC (ADT + ARPI: 48 [40%], ADT alone: 89 [55%], and ADT + NSAA: 64 [63%]). The IPTW-adjusted median (95% CI) time to mCRPC was not reached for the ADT + ARPI cohort, whereas median time to mCRPC was similar between the ADT + NSAA and ADT alone cohorts (14.4 [11.8─21.9] months and 14.6 [10.5─26.9] months, respectively). Patients treated with ADT + ARPI had a 60% lower risk of progression to mCRPC (IPTW-adjusted HR: 0.40 [95% CI: 0.27 to 0.59], *p* < 0.001) compared with patients treated with ADT alone (Fig. 2). In contrast, patients in the ADT + NSAA cohort had a similar risk of progression to mCRPC vs ADT alone (1.13 [0.83 to 1.56], *p* = 0.44). Due to a violation of the Cox proportional hazard model assumption, an alternative Cox model was created, which specifically compared ADT + ARPI vs ADT alone and yielded similar trends in the results (Supplementary Fig. 2).

### PSA reduction

**Fig. 3:**
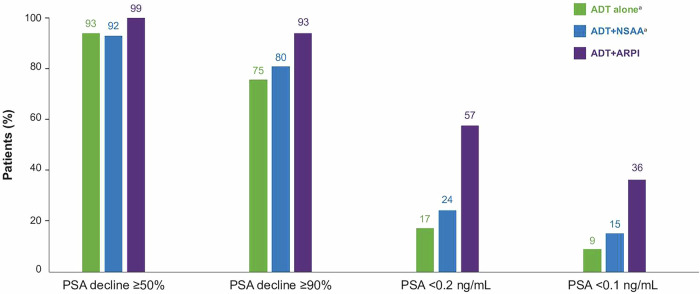
PSA response in patients with de novo mCSPC by first-line treatment. ADT androgen-deprivation therapy, ARPI androgen receptor pathway inhibitor, CI confidence interval, mCSPC metastatic castration-sensitive prostate cancer, NSAA nonsteroidal antiandrogen, PSA prostate-specific antigen. ^a^Three patients (two in the ADT alone cohort and one in the ADT + NSAA cohort) did not have a PSA test during first-line treatment.

**Fig. 4:**
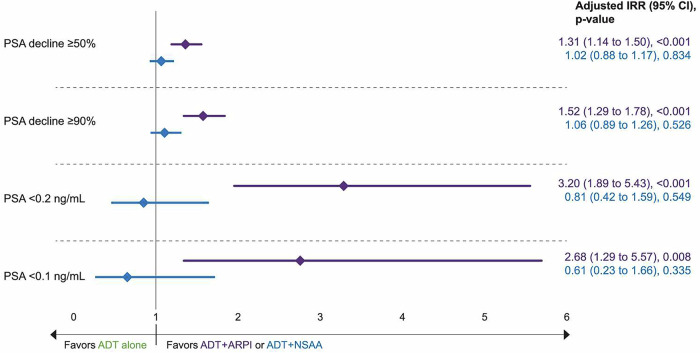
IPTW-adjusted comparison of the proportion of patients achieving PSA reduction by the first-line treatment. ADT androgen-deprivation therapy, ARPI androgen receptor pathway inhibitor, CI confidence interval, IPTW inverse probability of treatment weighting, IRR incidence rate ratio, NSAA nonsteroidal antiandrogen, PSA prostate-specific antigen.

Despite a higher median baseline PSA level, patients treated with ADT + ARPI demonstrated superiority in achieving PSA decline compared to those in the ADT alone cohort across multiple thresholds: PSA decline of ≥50% (99% vs 93%) and ≥90% (93% vs 75%), and to <0.2 ng/mL (57% vs 17%) and <0.1 ng/mL (36% vs 9%) (Fig. 3). In addition, compared with patients receiving ADT alone, those on ADT + ARPI were more likely to achieve PSA reduction ≥50% (IRR [95% CI]: 1.31 [1.14─1.50]), ≥90% (1.52 [1.29─1.78]), <0.2 ng/mL (3.20 [1.89─5.43]), and <0.1 ng/mL (2.68 [1.29─5.57]) (Fig. 4). The PSA decline for the ADT + NSAA and ADT alone cohorts were similar for all PSA outcomes in the adjusted analyses.

### Subsequent treatment after first-line therapy for de novo mCSPC

Of 384 de novo mCSPC patients, 12% (*n* = 45) received a second-line treatment while castration-sensitive. ADT + ARPI was the most common second-line therapy, followed by docetaxel and ADT + NSAA (Supplementary Fig. 3). The remaining 339 (88%) patients did not receive a second-line mCSPC treatment due to disease progression (46%), treatment discontinuation (24%), the end of the study period (17%), or death (14%).

Overall, 201 (52%) patients with de novo mCSPC developed castration resistance during follow-up and received a new regimen (first-line mCRPC treatment). ADT + ARPI was the most common first-line mCRPC treatment followed by ADT + NSAA (Supplementary Fig. 4).

## Discussion

In this retrospective cohort study of de novo mCSPC patients in the VA health care system, patients treated with first-line ADT intensification with ARPIs had significantly better survival outcomes compared with ADT alone. These findings resemble those from pivotal phase 3 trials and suggest that the survival benefits seen in mCSPC trials can be realized in clinical practice [10, 12–14, 16].

Compared with patients receiving ADT alone, patients receiving ADT + ARPI experienced a 39% lower risk of death in this study (HR: 0.61 [95% CI: 0.43 to 0.87]). Notably, the HR for OS in this real-world study is remarkably similar to that seen in clinical trials for ARPIs: abiraterone in STAMPEDE-A (0.60 [0.48−0.73]) [25] and LATITUDE (0.62 [0.51 to 0.76]) [13]; apalutamide in TITAN (HR 0.65 [0.53 to 0.79]) [16]; and enzalutamide in ENZAMET (0.60 [0.47 to 0.78]) [17] and ARCHES (0.66 [0.53 to 0.81]) [12]. Moreover, improvement in OS was accompanied by a delayed time to mCRPC in the ADT + ARPI cohort compared with ADT alone (HR [95% CI]: 0.40 [0.27 to 0.59]). This aligns with the longer time to mCRPC reported for enzalutamide in the ARCHES trial (0.28 [0.22 − 0.36]) [26] and apalutamide in the TITAN trial (0.34 [0.29 to 0.41]) [27]. The OS benefits also coincided with greater PSA decline with ADT + ARPI compared with ADT alone in this study, similar to what has been observed in previous phase 3 trials [28]. This is the first study to provide US real-world validation to clinical trial evidence, supporting the superiority of ADT + ARPI over ADT alone in patients with de novo mCSPC.

In this study, patients treated with an NSAA + ADT did not have significantly better OS, time to mCRPC, or PSA decline compared with ADT alone. A meta-analysis by the Prostate Cancer Trialists’ Collaborative Group also suggested that adding NSAA did not confer survival benefit during 1–2 years of follow-up and showed marginal superiority at 5 years [29]. Another VA-based real-world study found equal to marginally lower OS outcomes and similar time to mCRPC with ADT + NSAA vs ADT alone in patients with mCSPC [20]. Collectively, findings from this study, along with existing literature, suggest that TI with ARPI has marked survival benefits over ADT alone, whereas the addition of an NSAA to the ADT regimen likely yields, at best, a minimal benefit. Despite promising results from multiple well-designed clinical trials, use of TI for mCSPC has faced slow adoption and is underused, as shown in several real-world studies [20, 30–33]. Some of the key barriers to first-line TI for mCSPC reported by clinicians include poor trial knowledge, habit of not intensifying first-line, anticipated regret of intensifying early, costs, and reserving TI for later use [34]. Given the unequivocal benefits demonstrated in trials and confirmed in this real-world study, and despite the wide-spread use of ARPI in mCRPC for those who progressed in this study, it is crucial to enhance the incorporation of initial TI with ARPIs into clinical practice to improve mCSPC outcomes. This can be achieved by providing urologists and oncologists the resources identified to be most helpful to tackle this issue; decision support tools and clinical data summaries [12].

This study has some limitations. The analysis reflects patients with de novo mCSPC within the VA health care system and may not be generalizable to the civilian patient population. In addition, the number of patients for each treatment group was preset to reach certain quotas. Thus, the patient percentages in the three treatment groups do not necessarily reflect treatment patterns within the VA health care system. A race-based recruitment allocation was implemented to balance Black and White patients, but despite reviewing all eligible Black patients in each cohort, target inclusion numbers could not be achieved. We were also unable to evaluate toxicity between treatment groups in our analysis. However, a 2024 real-world evidence study conducted by Swami et al. using a claims database indicated that, among 4,145 patients with mCSPC, the cumulative incidences for seven out of 10 measured AEs were similar for patients treated with ADT + ARPIs vs those treated with ADT alone. Moreover, among patients treated with ADT + ARPIs, rates of most AEs were comparable to those seen among patients treated with ADT + NSAAs [35]. Finally, our analysis may be subject to residual confounding, as many variables that influence outcomes could not be captured in our study. The impact of this limitation on our results is unknown. However, this limitation is likely to be less impactful than in other similar studies, because, unlike many studies that rely on claims data, this study used a detailed chart review to confirm the de novo mCSPC diagnosis and assess treatments, and had detailed information of variables often lacking in claims data, such as PSA, Gleason score, and high- vs low-volume disease.

## Conclusions

First-line TI with ARPI showed significantly better survival outcomes compared with ADT alone in patients with de novo mCSPC, validating Level 1 evidence from trials and guidelines. Increased adoption of TI with ARPIs has the potential to improve OS for patients with de novo mCSPC.

## Supplementary information

Supplementary Information

## Data Availability

Researchers may request access to the data used to support this article by contacting the corresponding author.
