# Supplementary material for: Survival benefit associated with first-line androgen receptor pathway inhibitors for de novo metastatic castration-sensitive prostate cancer
Source: Prostate Cancer Prostatic Dis. 2025 Jul 25;29(1):167–74. doi: 10.1038/s41391-025-01000-8 (PMC12909124; doi:10.1038/s41391-025-01000-8)
Supplement: Supplementary file 1 — Supplementary Information [file 41391_2025_1000_MOESM1_ESM.docx]

**Title Page**

**Survival benefit associated with first-line androgen receptor pathway inhibitors for de novo metastatic castration-sensitive prostate cancer**

Stephen J. Freedland,^1,2*^Agnes Hong,^3^ Nader El-Chaar,^4^ Amanda M. De Hoedt,^1^ Janet Kim,^4^ Claire Evans,^1^ Joshua A. Parrish,^1^ Maelys Touya,^4^ Krishnan Ramaswamy,^3^ Lin Gu,^1^ Thomas J. Polascik^1,5^

*Author affiliations*

^1^Section of Urology, Durham VA Health Care System, Durham, NC, USA

^2^Department of Urology, Samuel Oschin Comprehensive Cancer Institute, Cedars-Sinai Medical Center, Los Angeles, CA, USA

^3^Pfizer Inc., New York, NY, USA

^4^Astellas Pharma Inc., Northbrook, IL, USA

^5^Duke Cancer Institute, Duke University School of Medicine, Durham, NC, USA

***Corresponding author**

Stephen J. Freedland

8631 S. Third St

Fourth Floor, Suite 430E

Los Angeles, CA 90048

Tel: 310-423-4700

Email: Stephen.freedland@cshs.org

**Supplementary methods**

Independent variables in the IPTW analysis: race (Black vs White), disease volume (high-volume vs low-volume per CHAARTED critera), age at index date (continuous), time from de novo mCSPC diagnosis to the index date (continuous), baseline lab values (including log-transformed PSA and alkline phosphatase [continuous]), the year of de novo mCSPC diagnosis (2018, 2019, or 2020), baseline chronic corticosteroid use (yes/no), body mass index (<25 kg/m^2^, 25–<30, or ≥30), Gleason score (Grade group 1 [Gleason 6 or fewer], Grade group 2 [Gleason 3 + 4 = 7], Grade group 3 [Gleason 4 + 3 = 7], Grade group 4 [Gleason 8], Grade group 5 [Gleason 9–10]), and CCI score (0, 1–2, 3–4, or 5+).

**Supplementary Table 1. Criteria for assessing progression to mCRPC and PSA response**

| **Response** | **Criteria** |
| --- | --- |
| Progression to mCRPC | - an increase in the PSA level of ≥25% and 2 ng/L from the nadir PSA evaluated from the index date. Each PSA value was required to be ≥14 days apart, and the lowest PSA value was to be ≥14 days after the index date. When there was no nadir value, evidence of CRPC was identified as an increase of PSA ≥25% and 2 ng/L from the baseline PSA value; - an increase in the number of metastases, absent CRPC by PSA rise, while castrate. Two scans from the same modality, one baseline and another showing progression, were required. The baseline was to be within 2 months of ADT initiation. Progression was defined as ≥2 new bone lesions or >20% increase in size for visceral and lymph node metastatic lesions. In case of positive baseline scans, no scan within 6 months of the ADT initiation was considered “progressive” due to tumor flare; - initial post-restart PSA at least as high as their final pre-restart PSA for patients who reinitiated ADT; - having evidence of cabazitaxel, mitoxantrone, radium-223, or sipuleucel-T after 90 days from the index date. |
| PSA response | - Nadir PSA was measured during first-line treatment and was defined as the lowest PSA value ≥14 days from the index date until the earliest of ADT treatment discontinuation date, CRPC date, death, or study end. - PSA response was measured in terms of the proportion of patients who achieved ≥50% and ≥90% decline from the baseline PSA value, or PSA values <0.2 ng/mL or <0.1 ng/mL. The baseline PSA was the last PSA value within 90 days of the index date. If ARPI/NSAA was started prior to the index date, the baseline PSA value was the last value prior to the start of ARPI/NSAA and within 120 days of the index date. |

Abbreviations: ADT, androgen-deprivation therapy; ARPI, androgen receptor pathway inhibitor; mCRPC, metastatic castration-resistant prostate cancer; NSAA, nonsteroidal antiandrogens; PSA, prostate-specific antigen.

**Supplementary Table 2. Unadjusted results for OS, time to mCRPC, PSA decline, and baseline PSA in patients with de novo mCSPC**

|  | ADT alone  (n = 163^a^) | ADT+NSAA  (n = 101^a^) | ADT+ARPI  (n = 120) |
| --- | --- | --- | --- |
| OS |  |  |  |
| OS (months), median (95% CI) | 28.1 (21.8 to 33.3) | 21.1 (18.1 to 30.4) | 49.0 (35.9 to NE) |
| Risk of death, HR (95% CI), P‑value | R | 1.17 (0.86 to 1.60), 0.317 | 0.54 (0.38 to 0.76), <0.001 |
| Progression to mCRPC |  |  |  |
| Time to mCRPC (months), median (95% CI) | 16.6 (12.4 to 26.9) | 13.6 (11.7 to 19.8) | NE (29.1 to NE) |
| Risk of progression to mCRPC, HR (95% CI), P-value | R | 1.18 (0.85 to 1.63),  0.315 | 0.51 (0.36 to 0.73),  <0.001 |
| Nadir PSA (ng/mL), median (IQR) | 2.6 (0.4, 16.1) | 1.5 (0.2, 10.6) | 0.1 (0.0, 0.9) |
| PSA decline, IRR (95% CI), P-value |  |  |  |
| PSA decline ≥50% | R | 1.04 (0.90 to 1.20), 0.62 | 1.39 (1.23 to 1.58),  <0.001 |
| PSA decline ≥90% | R | 1.06 (0.89 to 1.27), 0.509 | 1.65 (1.42 to 1.91),  <0.001 |
| PSA <0.2 ng/mL | R | 0.99 (0.53 to 1.86), 0.979 | 3.56 (2.23 to 5.68),  <0.001 |
| PSA <0.1 ng/mL | R | 0.91 (0.36 to 2.27), 0.835 | 3.23 (1.61 to 6.51), 0.001 |

Abbreviations: ADT, androgen-deprivation therapy; ARPI, androgen receptor pathway inhibitor; CI, confidence interval; HR, hazard ratio; IRR, incidence rate ratio; mCRPC, metastatic castration-resistant prostate cancer; NE, not estimable; NSAA, nonsteroidal antiandrogen; OS, overall survival; PSA, prostate-specific antigen; R, reference group.

^a^Three patients (two in the ADT alone cohort and one in the ADT+NSAA cohort) did not have a PSA test during first-line treatment.

**Supplementary Figure 1. Patient attrition flowchart**


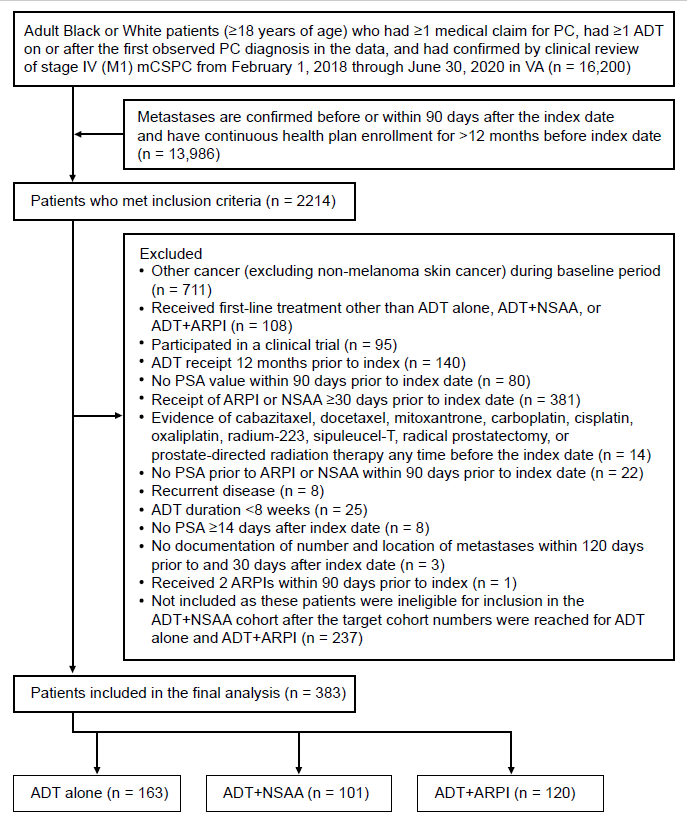


Abbreviations: ADT, androgen-deprivation therapy; ARPI, androgen receptor pathway inhibitor; mCSPC, metastatic castration-resistant prostate cancer; NSAA, nonsteroidal antiandrogen; PC, prostate cancer; PSA, prostate-specific antigen; VA, Veterans Affairs.

**Supplementary Figure 2. IPTW-adjusted time to mCRPC progression among patients with de novo mCSPC (Cox model without violation of proportional hazard assumptions)**


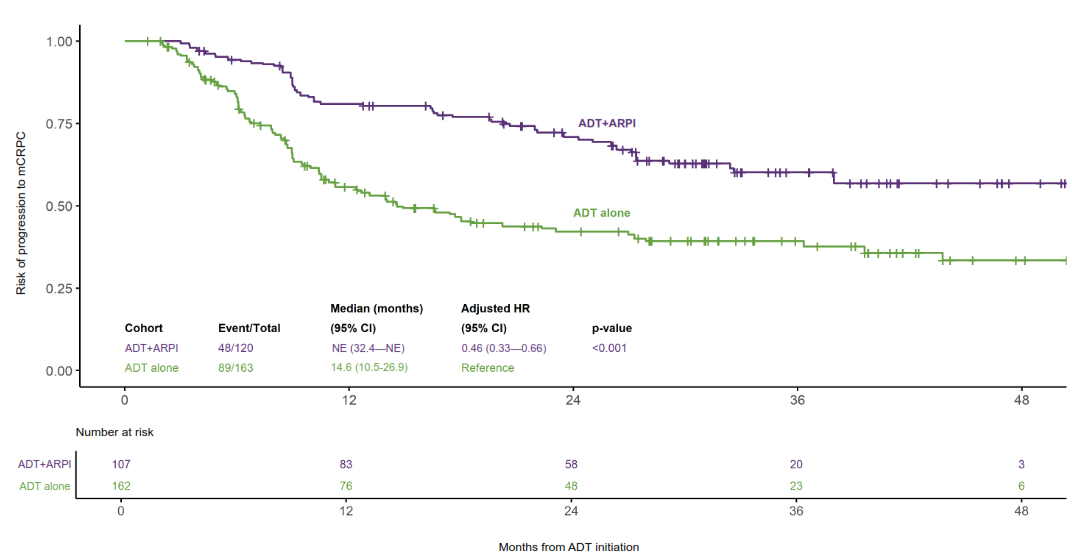


Abbreviations: ADT, androgen-deprivation therapy; ARPI, androgen receptor pathway inhibitor; IPTW, inverse probability of treatment weighting; mCRPC, metastatic castration-resistant prostate cancer; mCSPC, metastatic castration-sensitive prostate cancer; NE, not estimable; NSAA, nonsteroidal antiandrogen; PSA, prostate-specific antigen.

**Supplementary Figure 3. mCSPC treatment patterns among patients with de novo mCSPC**


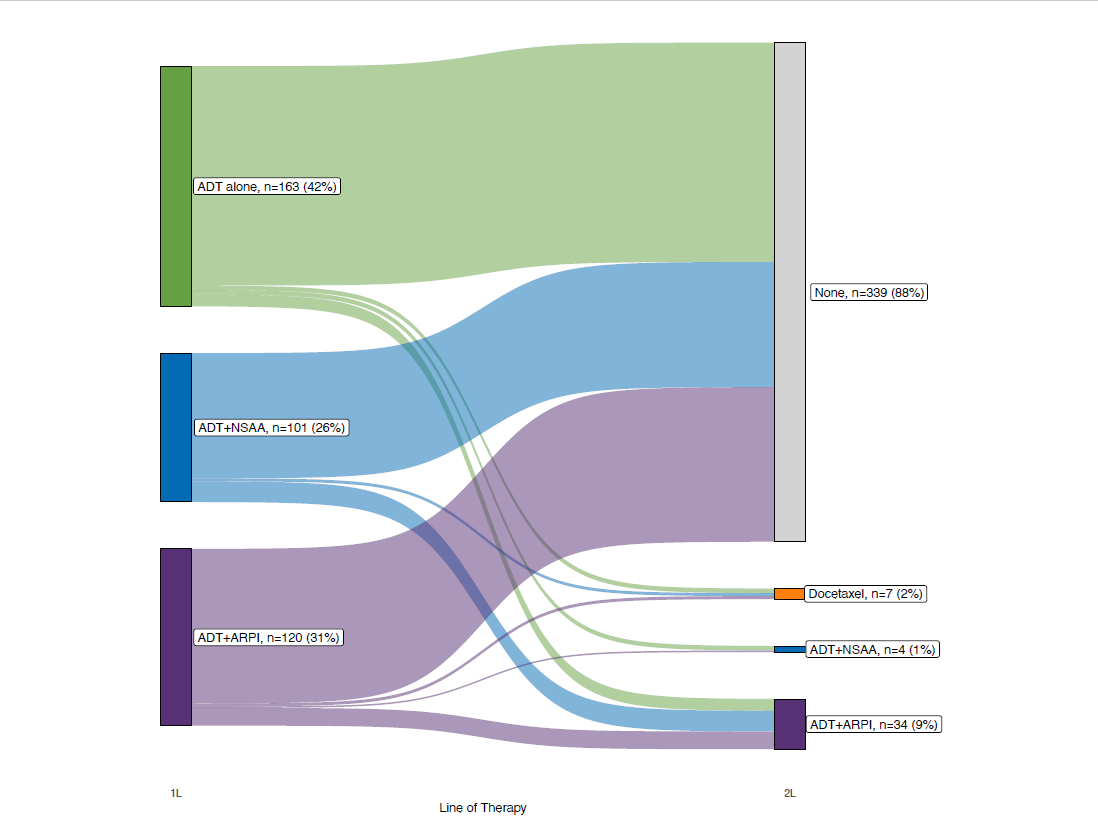


Abbreviations: ADT, androgen-deprivation therapy; LOT, line of therapy; mCSPC, metastatic castration-sensitive prostate cancer; NSAA, nonsteroidal antiandrogen.

None means no additional LOT observed, and it was due to progression, treatment discontinuation, end of the study period, or death.

**Supplementary Figure 4. Treatment patterns from last regimen observed while de novo mCSPC to mCRPC**


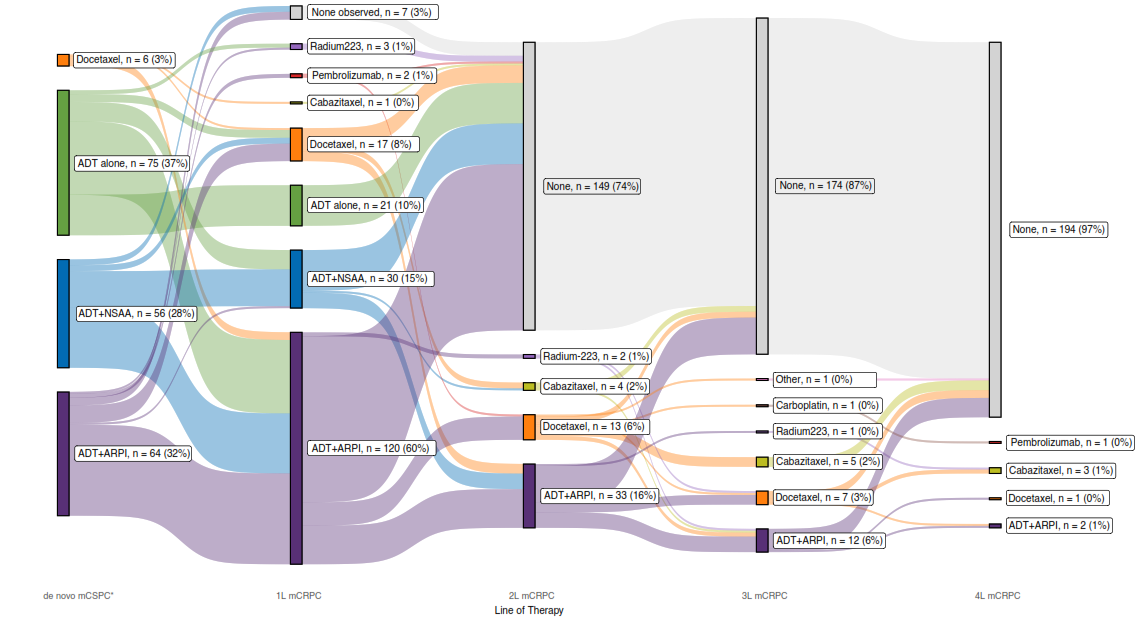


*This is the last mCSPC treatment observed prior to mCRPC.

Abbreviations: 1L, first line; 2L, second line; 3L, third line; 4L, fourth line; ADT, androgen-deprivation therapy; ARPI, androgen receptor pathway inhibitor; CRPC, castration-resistant prostate cancer; mCSPC, metastatic castration-sensitive prostate cancer; NSAA, nonsteroidal antiandrogen.
